# TRIP-Br2 promotes oncogenesis in nude mice and is frequently overexpressed in multiple human tumors

**DOI:** 10.1186/1479-5876-7-8

**Published:** 2009-01-20

**Authors:** Jit Kong Cheong, Lakshman Gunaratnam, Zhi Jiang Zang, Christopher M Yang, Xiaoming Sun, Susan L Nasr, Khe Guan Sim, Bee Keow Peh, Suhaimi Bin Abdul Rashid, Joseph V Bonventre, Manuel Salto-Tellez, Stephen I Hsu

**Affiliations:** 1 Renal Division and Department of Medicine, Brigham and Women's Hospital and Harvard Medical School, Boston, MA 02115, USA; 2 Department of Medicine, National University of Singapore and National University Hospital, 119074, Singapore; 3 Department of Pathology, National University of Singapore and National University Hospital, 119074, Singapore; 4 Division of Nephrology, Hypertension and Renal Transplantation, College of Medicine, University of Florida, 1600 SW Archer Road P.O. Box 100224, Gainesville, Florida 32610 USA

## Abstract

**Background:**

Members of the TRIP-Br/SERTAD family of mammalian transcriptional coregulators have recently been implicated in E2F-mediated cell cycle progression and tumorigenesis. We, herein, focus on the detailed functional characterization of the least understood member of the TRIP-Br/SERTAD protein family, TRIP-Br2 (SERTAD2).

**Methods:**

Oncogenic potential of TRIP-Br2 was demonstrated by (1) inoculation of NIH3T3 fibroblasts, which were engineered to stably overexpress ectopic TRIP-Br2, into athymic nude mice for tumor induction and (2) comprehensive immunohistochemical high-throughput screening of TRIP-Br2 protein expression in multiple human tumor cell lines and human tumor tissue microarrays (TMAs). Clinicopathologic analysis was conducted to assess the potential of TRIP-Br2 as a novel prognostic marker of human cancer. RNA interference of *TRIP-Br2 *expression in HCT-116 colorectal carcinoma cells was performed to determine the potential of TRIP-Br2 as a novel chemotherapeutic drug target.

**Results:**

Overexpression of TRIP-Br2 is sufficient to transform murine fibroblasts and promotes tumorigenesis in nude mice. The transformed phenotype is characterized by deregulation of the E2F/DP-transcriptional pathway through upregulation of the key E2F-responsive genes *CYCLIN E*, *CYCLIN A2*, *CDC6 *and *DHFR*. TRIP-Br2 is frequently overexpressed in both cancer cell lines and multiple human tumors. Clinicopathologic correlation indicates that overexpression of TRIP-Br2 in hepatocellular carcinoma is associated with a worse clinical outcome by Kaplan-Meier survival analysis. Small interfering RNA-mediated (siRNA) knockdown of TRIP-Br2 was sufficient to inhibit cell-autonomous growth of HCT-116 cells *in vitro*.

**Conclusion:**

This study identifies *TRIP-Br2 *as a *bona-fide *protooncogene and supports the potential for TRIP-Br2 as a novel prognostic marker and a chemotherapeutic drug target in human cancer.

## Background

Deregulation of E2F transcriptional activity due to alterations in the p16^INK4a^/cyclin D/RB pathway is a hallmark of many human cancers and more than half of all NCI-60 cell lines [1]. To date, the E2F family of proteins has been shown to be involved in the regulation of genes whose expression is pivotal for normal cell cycle progression and numerous other cellular processes such as DNA repair, programmed cell death and differentiation [2-4]. The TRIP-Br/SERTAD (henceforth referred to as TRIP-Br) family of novel mammalian transcriptional coregulators has recently been shown to modulate E2F-dependent transcriptional activities [5-7]. Family members include TRIP-Br1/p34^SEI-1^/SERTAD1/SEI-1 (henceforth referred to as TRIP-Br1), TRIP-Br2/SERTAD2/SEI-2 (henceforth referred to as TRIP-Br2), TRIP-Br3/HEPP/CDCA4/SEI-3 (henceforth referred to as TRIP-Br3), RBT1 (Replication Protein A Binding Transactivator 1)/SERTAD3 (henceforth referred to as RBT1) and the recently-identified SERTAD4 [8]. In addition, the TRIP-Br homolog in *Drosophila*, TARANIS (TARA), was identified in a screen for functional partners of the homeotic loci and was shown to represent a novel member of the trithorax group (trxG) of regulatory proteins [9].

Members of the TRIP-Br protein family possess three key regions that we have previously coined TRIP-homology domains (THD) [7]. THD1 contains a cyclin A-binding motif (including a conserved nuclear localization signal, KRK) at the amino terminal, followed by heptad repeats that have been shown to be essential for protein-protein interactions. THD2 consists of one or more PEST signals rich in proline, serine and threonine residues, while THD3 harbors a novel PHD zinc finger- and/or bromodomain-interacting motif and an acidic transactivation domain at its carboxyl-terminus. The heptad repeats in THD1 have been shown to be conserved in the TRIP-Br family and were renamed as the SERTA (**SE**I-1, **R**BT1 and **TA**RA) domain [9]. It has been further shown that most of the SERTA domain in TRIP-Br1 consists of a cyclin-dependent kinase 4 (CDK4)-binding site [10,11].

*TRIP-Br1 *and *RBT1 *have recently been shown to be localized in tandem within a 19q13 amplicon frequently found in human tumors, consistent with their putative role as oncogenes that promote tumor growth [5]. Indeed, cytogenetic studies have revealed a gain of chromosomal region 19q13.1-13.2 in more than 30% of ovarian carcinomas [12,13] as well as a variety of other tumors including pancreatic carcinomas [14] and lung cancers [15]. Although *TRIP-Br1 *has been further demonstrated to be amplified and overexpressed in several ovarian cancer cell lines as well as in ovarian carcinomas [16], the association of *RBT1 *amplification to human cancers remains elusive. As a proof-of-principle that at least a subset of the *TRIP-Br *gene family consists of novel protooncogenes that play important roles in cellular proliferation and human cancer, the knockdown of *TRIP-Br1 *or *RBT1 *in cultured cell lines has been shown to reduce cell growth and colony formation [5,17,18]. Apart from their role as coactivators in the stimulation of E2F-dependent transcription, the corepressor function of TRIP-Br proteins has also been described. Overexpression of TRIP-Br1 has been found to suppress CREB-mediated transcription and this suppression could be overcome by ectopic overexpression of CBP [19]. In addition, *TRIP-Br3 *has been recently identified as a novel E2F-responsive gene and as a repressor of E2F-dependent transcriptional activation [6].

While most of the TRIP-Br family members have recently been extensively characterized and shown to be involved in a variety of important cellular processes including E2F-mediated cell cycle progression, p53-dependent stress response and cancer pathogenesis [6,7,9,11,18,20-22], the physiological role of TRIP-Br2 in mammalian cells remains poorly understood and its direct link to cancer pathogenesis has not been established. We previously reported that transcriptional downregulation of *TRIP-Br2 *in primary cell lines, achieved through DNA enzyme knockdown or global knockout strategies, results in cellular proliferation arrest [17]. In the present study, we have validated the oncogenic potential of TRIP-Br2. Overexpression of TRIP-Br2 resulted in the upregulation of E2F-mediated transcription, the transformation of NIH3T3 fibroblasts and the promotion of tumor growth in athymic nude mice. We further performed high-throughput expression profiling of TRIP-Br2 in comprehensive human tumor tissue microarrays and showed that TRIP-Br2 is frequently overexpressed in cancer.

## Methods

### Analysis of TRIP-Br2 gene structural organization, prediction of TRIP-Br2 protein subcellular localization and in silico profiling of TRIP-Br2 gene expression

The gene structural organization of human *TRIP-Br2 *was analyzed by NCBI Entrez Gene, NCBI AceView and BLAST/ClustalW . The PSORT II analysis software  was used to predict the subcellular localization of TRIP-Br2 proteins. The GNF SymAtlas v 1.2.4 (Novartis, ) human microarray database was interrogated to determine the *in silico *gene expression profiling of *TRIP-Br2 *across all human tissues. The NCBI symbol SERTAD2 was used in the query of the GNF SymAtlas database. The median (med) was calculated based on expression of *TRIP-Br2 *across all human tissues; med × 3: 3-fold more than the median; med × 10: 10-fold more than the median. *In silico TRIP-Br2 *expression, (χ), across all human tissues was scored via the following scheme: +: (χ) ≤ median; ++: median < (χ) ≤ med × 3, +++: med × 3 < (χ) ≤ med × 10, ++++: med × 10 < (χ).

### Cell culture and reagents

NIH3T3 mouse primary fibroblasts, WI38 human primary lung fibroblasts, U2OS human osteosarcoma cells, PC3 human prostate adenocarcinoma cells, 769-P human renal adenocarcinoma cells, HCT-116 human colorectal carcinoma cells, HepG2 human hepatocellular carcinoma cells and MCF-7 human breast carcinoma cells were purchased from American Type Culture Collection (Manassas, VA). All cell lines were cultured in DMEM supplemented with 10% FBS and maintained at 37°C in a 5% CO_2 _environment. Rabbit anti-TRIP-Br2 polyclonal antibodies were generated as previously described [23] and used in Western blot, immunocytochemical and immunohistochemical analyses. All other antibodies used in Western blot analyses were purchased from Santa Cruz Biotechnology, Inc. (Santa Cruz, CA). They include anti-HA (sc-805), anti-cyclin E (sc-481) and anti-β-tubulin (sc-5274). The use of expression plasmids pcDNA3.1 (Invitrogen, Carlsbad, CA) and pcDNA3.1-*TRIP-Br1-HA *have been previously described [7]. The nucleotide sequence of human *TRIP-Br2 *(*hTRIP-Br2*) was obtained from NCBI PubMed (GenBank™ accession no. BC101639) and used as the template in the design of *hTRIP-Br2*-specific primers for the construction of C-terminal HA-tagged hTRIP-Br2 expression plasmid (Additional File 1).

### Generation of cells stably expressing TRIP-Br2

NIH3T3 fibroblasts were transfected with the empty vector pcDNA3.1 as a control or with the expression vectors pcDNA3.1-*TRIP-Br1-HA *or pcDNA3.1-*TRIP-Br2-HA *using FuGENE 6 Transfection Reagent (Roche Diagnostics Co., Mannheim, Germany) in accordance with the manufacturer's instructions. Stable clones were selected using Geneticin (Invitrogen, Carlsbad, CA) at a concentration of 750 μg/ml. Expression levels of the carboxyl terminal HA-tagged TRIP-Br1 and TRIP-Br2 in each respective clone were determined by Western blot analysis.

### Serum deprivation, Bromodeoxyuridine (BrdU) labeling and flow cytometric DNA content analysis

NIH3T3^vector-only^, NIH3T3^TRIP-Br1-HA ^and NIH3T3^TRIP-Br2-HA ^fibroblasts were cultured in 96-well plates (for BrdU) or 100 mm culture dishes (for flow cytometry) in DMEM supplemented with 0.2% FBS and were maintained for 72 h at 37°C in a 5% CO_2 _environment. BrdU incorporation was monitored using a cell proliferation/colorimetric ELISA assay according to the manufacturer's instructions (Boehringer Mannheim, Mannheim, Germany). Flow cytometry was performed using a FACScan flow cytometer (Becton Dickinson, Franklin Lakes, NJ) at a wavelength of 488 nm.

### Soft agar colony formation and tumor induction assays

Soft agar assays were used to assess anchorage-independent growth of NIH3T3 cells as previously described [24]. For tumor induction assays, athymic nude mice (nu/nu) purchased from Charles River Laboratories, Inc. (Wilmington, MA) were kept under SPF conditions and used under protocol #06-231, which was approved by the Harvard Institutional Animal Care and Use Committee (IACUC) and the Harvard Committee on Microbiological Safety (COMS). 5 × 10^6 ^NIH3T3^vector-only ^or NIH3T3^TRIP-Br2-HA ^fibroblasts were injected subcutaneously into 6-week-old athymic nude mice (n = 4 for each group). On day 13 post-injection, the mice were examined for tumor formation. Tumor dimensions were measured every 2 days from day 13 until day 25 post-injection, at the end of which time both groups were sacrificed and all tumors were harvested for histological, immunohistochemical and Western blot analyses. The experiment was repeated by injection of new NIH3T3^vector-only ^or NIH3T3^TRIP-Br2-HA ^clones into new groups of 6-week-old athymic nude mice (n = 4). The penetrance of tumor induction from subcutaneous injection of NIH3T3^vector-only ^or NIH3T3^TRIP-Br2-HA ^into these athymic nude mice was 0% and 100%, respectively. Tumor ellipsoid volume was estimated using the formulae previously described [25].

### Semi-quantitative RT-PCR analyses

Total RNA was isolated from serum-deprived NIH3T3^vector-only^, NIH3T3^TRIP-Br1-HA ^and NIH3T3^TRIP-Br2-HA ^fibroblasts using the TRIZOL^® ^Reagent (Invitrogen, Carlsbad, CA). Total RNA (3 μg) was reverse transcribed using the ABI High Capacity cDNA Archive Kit (Applied Biosystems, Foster City, CA) according to the manufacturer's instructions. Polymerase Chain Reactions (PCR) were performed on 1 μl cDNA samples in the presence of 10 mM deoxyribonucleotide triphosphates (dNTPs) and 10 μM of specific primer pairs in a total reaction volume of 20 μl. PCR was performed as follows: 20 cycles of denaturation (94°C, 30 sec), annealing (51°C, 30 sec) and extension (72°C, 1 minute) with a 2-minute initial denaturation step at 94°C and a 3-minute terminal polishing step at 72°C. The primer sequences used for RT-PCR are available upon request.

### Subcellular fractionation, denaturing SDS-PAGE and Western blotting

Subcellular fractionation of the cells was performed using the NE-PER Nuclear and Cytoplasmic Extraction Reagents Kit (Pierce Biotechnology, Inc., Rockford, IL) according to the manufacturer's instructions. Proteins from whole-cell lysates were resolved using standard denaturing polyacrylamide gel electrophoresis and immunostained as described previously [7].

### Tissue microarray (TMA) construction, immunohistochemistry and immunocytochemistry

Multiple TMA slides were obtained from the Department of Pathology TMA Program at the National University of Singapore, in compliance with Institutional Review Board approval (IRB 05-017). These tumor TMAs were constructed as previously described [26-29] and represented samples from the following human tumor types that occur in a broad range of organs: prostate carcinoma, squamous cell lung carcinoma, lung adenocarcinoma, breast carcinoma, gastrointestinal stromal tumor, ovarian cystadenocarcinoma, colorectal carcinoma, basal cell carcinoma, renal cell carcinoma, osteosarcoma, hepatocellular carcinoma. Antigens were retrieved from the tissues using a microwave histoprocessor (Milestone, Shelton, CT) and DAKO pH 6.0 citrate buffer (DAKO, Via Real Carpinteria, CA). Immunohistochemical staining was performed on paraffin-embedded tissue sections using the DAKO Envision kit (DAKO) and the rabbit anti-TRIP-Br2 antibody or its pre-immune serum control at a concentration of 1:300. Staining was visualized using a Leica DM LB2 microscope. The intensity of TRIP-Br2 expression by immunostaining in the tumor TMAs was scored independently by three research pathologists in a double-blinded manner. For immunocytochemistry, cells were grown to 80% confluence on coverslips, washed three times with PBS, fixed in pre-chilled 4% paraformaldehyde for 20 minutes, and permeabilized in 0.1% Triton-X for 10 minutes. Primary immunostaining with rabbit anti-TRIP-Br2 antibody (1:4000) was performed at room temperature for 1 h. Pre-immune rabbit serum was used as a negative control for the primary immunostaining of cells. Secondary immunostaining with goat anti-rabbit-FITC antibodies (sc-2012, Santa Cruz Biotechnology, Inc., Santa Cruz, CA) was performed at room temperature for 1 h, following 3 washes with PBS at the end of primary immunostaining. Cellular DNA was subsequently counterstained with DAPI. Staining was visualized and photographed using a Nikon Eclipse E1000 fluorescence microscope.

### RNA interference of TRIP-Br2 expression

5 × 10^4 ^HCT-116 cells were plated in 12-well plates and transfected with Cy3-labeled oligomer, scrambled siRNA (negative control) or three different *TRIP-Br2*-specific siRNAs at the dose of 4 picomoles (pmol) or 40 pmol (in 1 ml of DMEM supplemented with 10% FBS) respectively (TriFECTa™ kit, IDT, Coralville, IA) using Lipofactamine™ Transfection Reagent (Invitrogen, Carlsbad, CA), in accordance with the manufacturer's instructions. Twenty-four hours post-transfection, these cells were cultured in serum-free DMEM and maintained at 37°C in a 5% CO_2 _environment for 72 h. HCT-116 cells that were not subjected to transfection reagent treatment were included as controls. Cells in colony forming assays were stained with 0.4% Giemsa stain as previously described [23]. The dye in these cells was subsequently eluted with 1% SDS and quantitated using a spectrophotometer at a wavelength of 595 nm. A standard curve was plotted using OD readings taken from dye-eluted HCT-116 cells that were plated at pre-determined cell densities.

### Statistical analysis

Survival curves for various patient cohorts were estimated according to the method of Kaplan and Meier, and curves were compared using the generalized Wilcoxon's test. The log-rank test was used to assess the strength of association between survival time and single variables corresponding to factors thought to be prognostic for survival.

## Results

### TRIP-Br2, a novel proliferation marker, is highly expressed in human lymphohematopoietic cell lineages

**Figure 1:**
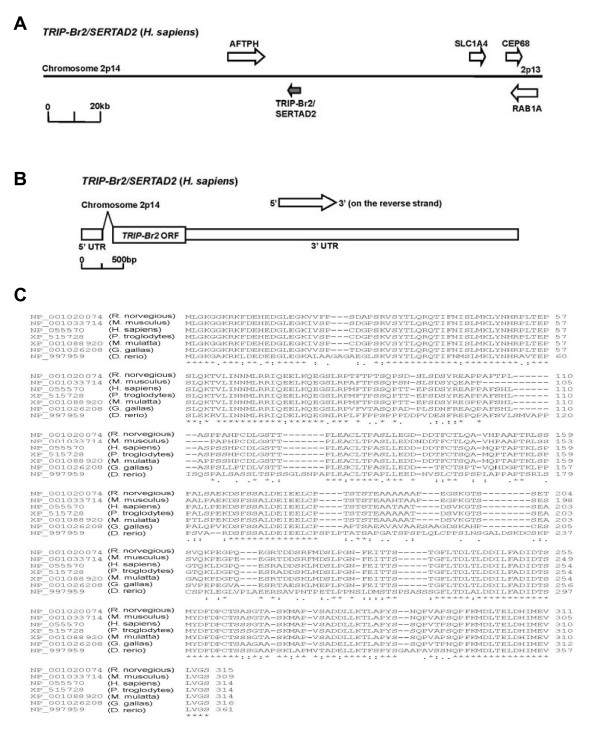
**Gene structural organization of human *TRIP-Br2***. (A) *TRIP-Br2 *is localized at chromosome 2p14 of the human genome (B) *TRIP-Br2 *consists of two exons that are separated by an intron encoding splice donor-acceptor (GT-AG) sequences at either end. (C) Multiple sequence alignment of TRIP-Br2 proteins from widely divergent species by BLAST/ClustalW analyses.

The *TRIP-Br2 *gene locus is approximately 22.3 kb long and is localized at the poorly-characterized chromosome 2p14 region of the human genome (position 64734550 bp to 64712250 bp; reverse strand) (Figure 1A). The precursor of *TRIP-Br2 *mRNA is approximately 5556 bp in length and consists of two exons that are separated by a long intron (Figure 1B). The intron encodes a splice donor (GT) and a splice acceptor (AG) at either end, respectively. The 297 bp-long 5' untranslated region (UTR) resides in exon 1 of *TRIP-Br2*. It contains an in-frame stop signal that is 6 bp prior to the 945 bp-long coding sequence of *TRIP-Br2*, which is localized in exon 2. The 3' UTR of *TRIP-Br2 *spans a region of approximately 4314 bp, followed by a standard AATAAA polyadenylation signal. Due to the lack of other splice donor-acceptor sites, transcription of the human *TRIP-Br2 *gene is predicted to yield only one mRNA transcript that encodes a 314 amino acid protein. We studied the primary protein sequence of human TRIP-Br2 by BLAST/ClustalW analyses and found that TRIP-Br2 is highly conserved in widely divergent species, such as chimpanzee (99%), rhesus monkey (97%), rat (86.9%), mouse (88.3%), chicken (81.4%) and zebrafish (67.1%) (Figure 1C). Furthermore, based on the PSORT II analysis, the subcellular localization of TRIP-Br2 protein is predicted to be predominantly in the nucleus (69%), with scant presence in the mitochondria (17%), in the cytoplasm (4%), in the vacuoles (4%) or in vesicles of the secretory system (4%).

In order to investigate the biological significance of TRIP-Br2 in humans, we first performed *in silico *gene expression profiling of *TRIP-Br2 *using a comprehensive web-based human microarray database, GNF SymAtlas v 1.2.4 (Novartis, ). As compared to other tissues/cell types, *TRIP-Br2 *is highly expressed in bone marrow, the thymus, the tonsil and smooth muscle. It is also highly expressed in lymphohematopoietic cell lineages, particularly in BDCA4+ dendritic cells, CD34+ cells (bone marrow hematopoietic stem cells), CD71+ early erythroid cells, B lymphoblasts, CD4+ T cells, CD8+ T cells, CD19+ B cells, CD56+ NK cells and CD33+ myeloid cells (Table 1). As these cell types are highly proliferative, we postulated that TRIP-Br2 plays an important role in cellular proliferation and/or tumor progression. This is supported in part by our previous observation that ablation of *TRIP-Br2 *resulted in cellular proliferation arrest in primary cells, which was associated with downregulation of a subset of E2F-responsive genes such as *CYCLIN E *[17].

**Table 1:** *TRIP-Br2 *expression profiling in human tissues by interrogation of the Novartis GNF SymAtlas v1.2.4 microarray database

**Human tissues**	***TRIP-Br2 *gene expression**
Bronchial epithelial cells	++
Lung	+
Whole brain	+
Bone marrow*	+++
Thymus*	++++
Lymph node	++
Tonsil*	+++
Heart	+
Liver	+
Kidney	+
Skin	+
Pancreas	+
Skeletal muscle	+
Cardiac myocytes	+
Smooth muscle*	+++
Placenta*	+++
Prostate	++
Uterus	++
Ovary	+
Testis	+

Lymphohematopoietic cell lineages	
BM-CD34+ cells*	++++
BM-CD71+ early erythroid cells*	++++
BM-CD105+ endothelial cells*	+++
BM-CD33+ myeloid cells*	++++
PB-CD56+ NK cells*	++++
PB-BDCA4+ dendritic cells*	+++
PB-CD14+ monocytes*	++++
PB-CD19+ B cells*	++++
B lymphoblasts*	++++
CD4+ T cells*	+++
CD8+ T cells*	+++

The median (med) was calculated based on expression of *TRIP-Br2 *across all human tissues; med × 3: 3-fold more than the median; med × 10: 10-fold more than the median. *In silico TRIP-Br2 *expression, (χ), across all human tissues was scored via the following scheme: +: (χ) ≤ median; ++: median < (χ) ≤ med × 3, +++: med × 3 <(χ) ≤ med × 10, ++++: med × 10 <(χ). *TRIP-Br2 *is found to be highly expressed in human tissues such as bone marrow, thymus, tonsil and smooth muscle. *TRIP-Br2 *is also highly expressed in the highly proliferative lymphohematopoietic cell lineages. *Denotes high *TRIP-Br2 *expression in these cells and tissues. BM: Bone marrow-derived; PB: Peripheral blood-derived.

### TRIP-Br2 overexpression transforms murine fibroblasts by upregulation of E2F/DP-mediated transcription

**Figure 2:**
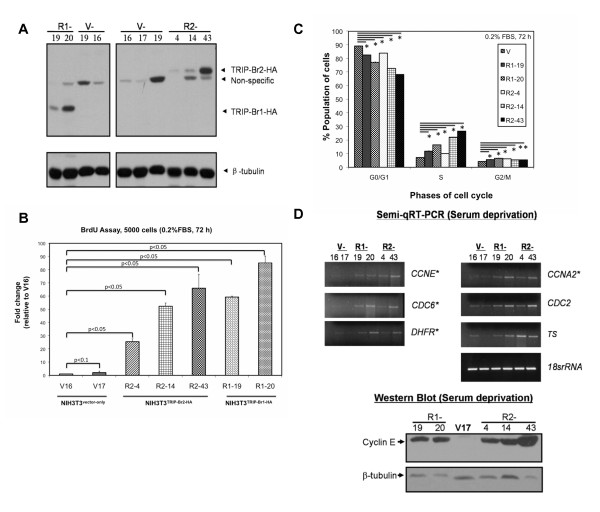
**TRIP-Br-overexpressing-NIH3T3 fibroblasts proliferate in the absence of mitogenic stimulation as a result of deregulation of the RB/E2F/DP1 transcriptional pathway**. V: Vector-only clones, R1: TRIP-Br1-HA-overexpressing clones, R2: TRIP-Br2-HA-overexpressing clones. β-tubulin was used as a loading control. (A) NIH3T3 fibroblasts were transfected with pCDNA3.1 vector (NIH3T3^vector-only^), pCDNA3.1-*TRIP-Br1-HA *(NIH3T3^TRIP-Br1-HA^) or pCDNA3.1-*TRIP-Br2-HA *(NIH3T3^TRIP-Br2-HA^), selected by G418, and analyzed by immunoblotting using an anti-HA antibody. Data was obtained from three independent experiments that were performed in triplicates. (B) NIH3T3^vector-only^, NIH3T3^TRIP-Br1-HA ^and NIH3T3^TRIP-Br2-HA ^fibroblasts were cultured in 96-well plates in DMEM supplemented with 0.2% FBS and were maintained for 72 h at 37°C in a 5% CO_2 _environment. BrdU incorporation was monitored using a cell proliferation/colorimetric ELISA assay. The fold increase in BrdU incorporation of all clones was calculated relative to that of V16, which was set arbitrarily to 1.0. The error bars represent the standard deviations of three independent experiments performed in triplicates. A Student's t-test was performed and the respective p-values were indicated in the bar chart. (C) Upon serum deprivation, S phase cell counts were significantly higher in the TRIP-Br-overexpressing NIH3T3 clones than the vector-only control. The results shown represent the mean ± SD for each independent R1 (-19 and -20) and R2 clone (-4, 14, 43), compared to all V clones combined (-16, -17, -19), and incorporate data from 3 independent experiments performed in triplicate. A Student's t-test was performed; *indicates p-value < 0.001; **indicates p-value < 0.01 for the comparison of NIH3T3^vector-only ^and NIH3T3^TRIP-Br1-HA ^or NIH3T3^TRIP-Br2-HA ^cells. (D) *Upper panel: *Semi-quantitative RT-PCR analyses revealed up-regulation of *CYCLIN E *(*CCNE*), *CYCLIN A2 *(*CCNA2*), *CDC6 *and *DHFR *in serum-deprived NIH3T3^TRIP-Br1-HA ^and NIH3T3^TRIP-Br2-HA ^fibroblasts. *TS*: *Thymidylate synthase*; *18srRNA *was used as a loading control. Data was obtained from three independent experiments that were performed in triplicates. *Lower panel: *Western blot analyses showed an increase in cyclin E in serum-deprived NIH3T3^TRIP-Br1-HA ^and NIH3T3^TRIP-Br2-HA ^fibroblasts when these cells were immunostained with anti-cyclin E antibodies. Data was obtained from three independent experiments that were performed in triplicates.

To validate the protooncogenic role of TRIP-Br2 in cell cycle regulation and tumorigenesis, we stably overexpressed C-terminal HA-tagged-TRIP-Br2 in NIH3T3 fibroblasts (NIH3T3^TRIP-Br2-HA^; Figure 2A). Although TRIP-Br1 overexpression has been recently shown to transform NIH3T3 fibroblasts, the underlying molecular mechanism of cellular transformation by TRIP-Br1 remains elusive. Thus, we also stably overexpressed carboxyl-terminal HA-tagged-TRIP-Br1 in NIH3T3 fibroblasts (NIH3T3^TRIP-Br1-HA^) and investigated the mechanism(s) by which TRIP-Br1 and TRIP-Br2 facilitate cellular transformation. Overexpression of TRIP-Br1-HA or TRIP-Br2-HA in NIH3T3 fibroblasts conferred the ability to proliferate under low serum concentrations, possibly by enhancing DNA synthesis (Figure 2B). Flow cytometric DNA analysis revealed significantly higher proportions of NIH3T3^TRIP-Br1-HA ^and NIH3T3^TRIP-Br2-HA ^fibroblasts in S phase of the cell cycle as compared to the NIH3T3^vector-only ^control, despite serum deprivation (Figure 2C). As TRIP-Br proteins have been shown to regulate E2F/DP-mediated transcriptional activities [7], we screened these serum-deprived NIH3T3 fibroblasts for a panel of E2F-responsive cell cycle regulators that govern cell cycle progression. Elevated levels of a subset of these E2F-responsive cell cycle regulators comprised of *CYCLIN E *(*CCNE*), *CYCLIN A2 *(*CCNA2*), *CDC6 *and *DHFR*, were found in serum-deprived fibroblasts that stably overexpress TRIP-Br proteins (Figure 2D, *upper panel*). Notably, in serum-deprived NIH3T3 cells that stably overexpress TRIP-Br1-HA or TRIP-Br2-HA, we observed a concomitant increase in cyclin E expression (Figure 2D, *lower panel*). This is consistent with our previous observation that cyclin E was downregulated following ablation of *TRIP-Br1 *or *TRIP-Br2*. Hence, our data suggest that *CYCLIN E *may be a TRIP-Br1- and TRIP-Br2-coregulated gene.

### TRIP-Br2 overexpression confers anchorage-independent growth in soft agar and promotes tumor growth in athymic nude mice

**Figure 3:**
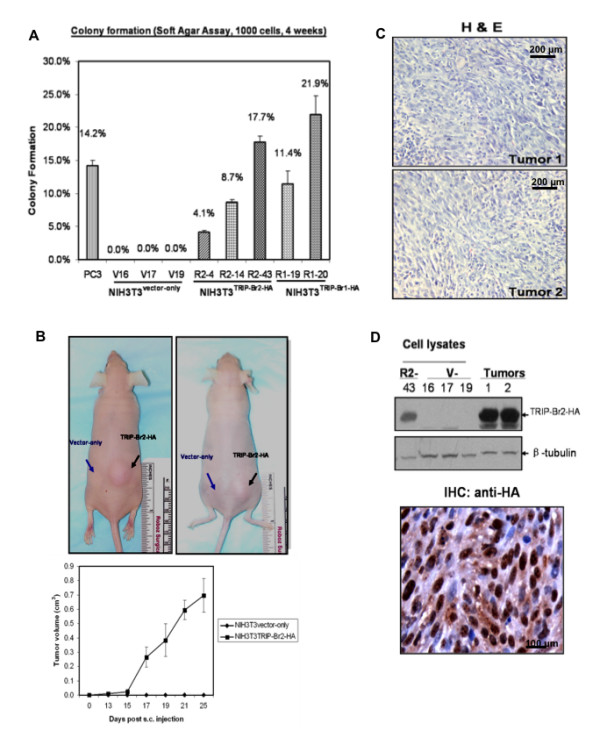
**Overexpression of TRIP-Br2-HA confers anchorage-independent growth on soft agar and induces tumors in nude mice (nu/nu)**. (A) Anchorage-independent growth of NIH3T3^vector-only^, NIH3T3^TRIP-Br1-HA ^and NIH3T3^TRIP-Br2-HA ^was assessed by colony formation in soft agar. The error bars represent the standard deviations of three independent experiments performed in triplicates. PC3 cells were used as a positive control. V: Vector-only clones; R1: TRIP-Br1-HA-overexpressing clones; R2: TRIP-Br2-HA-overexpressing clones. (B) *Upper panel*: Results of a representative experiment in which NIH3T3^TRIP-Br2-HA ^and NIH3T3^vector-only ^fibroblasts were subcutaneously injected into the left and right flanks of nude mice, respectively. *Lower panel*: Average tumor ellipsoid volume over 25 days post-subcutaneous injection was calculated, and the animals were subsequently sacrificed. (C) Histological analyses of excised tumors indicated the presence of fibrosarcomas. (D) Western blot (*Upper panel*) and immunohistochemical analyses (*Lower panel*) of excised tumors showed expression of TRIP-Br2-HA. Immunopositive staining for TRIP-Br2-HA is represented by the brown color against the hematoxylin (blue) counterstain. Data was obtained from three independent experiments that were performed in triplicates.

Next, we evaluated the oncogenic potential of TRIP-Br2 by assessing anchorage-independent growth of these NIH3T3^TRIP-Br2-HA ^fibroblasts in soft agar (Figure 3A). As many as 17.7% of the seeded NIH3T3^TRIP-Br2-HA ^fibroblasts formed colonies at 4 weeks post-plating, while NIH3T3^vector-only ^fibroblasts were incapable of anchorage-independent growth in soft agar. PC3 cells and NIH3T3^TRIP-Br1-HA ^fibroblasts were used as positive controls in this assay.

In addition, we validated the tumorigenic potential of TRIP-Br2 by an *in vivo *tumor formation assay. One inoculum (5 × 10^6 ^cells) of either NIH3T3^vector-only ^or NIH3T3^TRIP-Br2-HA ^fibroblasts (from one representative clone each) was injected subcutaneously into the lower flanks of athymic nude mice (n = 4). This experiment was repeated at least twice by subcutaneous injection of a different clone of either NIH3T3^vector-only ^or NIH3T3^TRIP-Br2-HA ^fibroblasts into other groups of four athymic nude mice. Data from two mice from a representative experiment are shown in Figure 3B (*Upper panel*). All sites injected with NIH3T3^TRIP-Br2-HA ^fibroblasts developed a tumor, which was typically ~0.7 cm^3 ^(data derived from one tumor induction assay, n = 4) at day 25 post-injection (Figure 2B, *lower panel*). Tumors derived from NIH3T3^TRIP-Br2-HA ^fibroblasts were histologically fibrosarcomas (Figure 3C). Western blot analyses of tumor extracts (Figure 3D, *upper panel*) as well as HA-immunostaining of paraffin-embedded tumor sections (Figure 3D, *lower panel*) indicated the presence of the transgene product TRIP-Br2-HA.

### TRIP-Br2 expression is dysregulated in many human cancer cell lines

**Figure 4:**
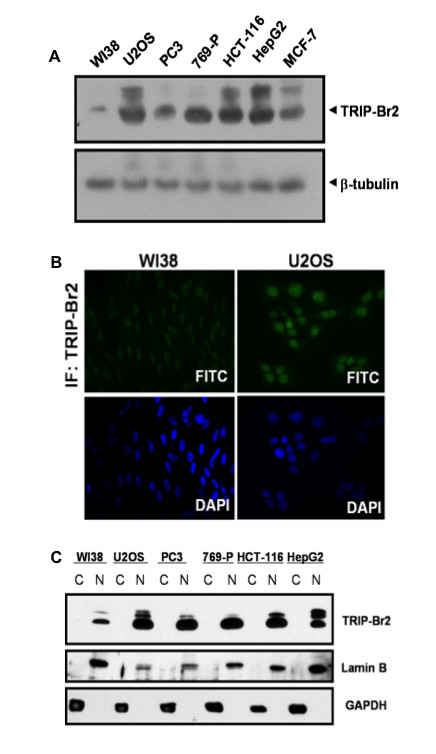
**TRIP-Br2 expression is dysregulated in many human cancer cell lines**. (A) Western blot analyses revealed overexpression of TRIP-Br2 in the human cancer cell lines U2OS, PC3, 769-P, HCT-116, HepG2 and MCF-7. Data was obtained from three independent experiments that were performed in triplicates. (B) Immunocytochemical analyses showed that TRIP-Br2 is found predominantly in both the nuclei of WI38 and U2OS cells. Cellular DNA was counterstained with DAPI (blue). (C) Subcellular fractionation analyses revealed that TRIP-Br2 is overexpressed and preferentially localized to the nuclei of U2OS, PC3, 769-P, HCT-116 and HepG2 cells. Data was obtained from three independent experiments that were performed in triplicates.

Given that overexpression of TRIP-Br2 alone was sufficient to transform NIH3T3 fibroblasts, we hypothesized that expression of TRIP-Br2 may be dysregulated and contribute to oncogenesis in human cancer. We screened normal and cancer cell lines for TRIP-Br2 expression using rabbit anti-TRIP-Br2 polyclonal antibodies and found that TRIP-Br2 was overexpressed in human cancer cell lines U2OS, PC3, 769-P, HCT-116, HepG and MCF-7 cells, but not in WI38 diploid fibroblasts (Figure 4A). The higher molecular weight endogenous species of TRIP-Br2 observed in Figure 4A (and 4C below) are specific bands that we have observed in only some human cancer cell lines, associated with the use of the rabbit polyclonal anti-TRIP-Br2 for immunoblot analysis [23].

We next sought to identify the cellular role(s) of TRIP-Br2 by investigating its localization in WI38 and U2OS cells. Using rabbit anti-TRIP-Br2 polyclonal antibodies, we first demonstrated by immunocytochemistry that TRIP-Br2 was predominantly localized to the nuclei of WI38 and U2OS cells (Figure 4B), with scant cytoplasmic expression. This is in agreement with our earlier PSORT II prediction of TRIP-Br2 subcellular localization and a recent observation made by Lai and coworkers [30]. As compared to WI38 cells, TRIP-Br2 was clearly overexpressed in the nuclei of U2OS cells. Our data from subcellular fractionation analysis is consistent with this observation. TRIP-Br2 was overexpressed and predominantly localized to the nuclear fractions of U2OS cells as well as other human cancer cell lines such as PC3, 769-P, HCT-116 and HepG2 (Figure 4C), suggesting that TRIP-Br2 expression and localization might be dysregulated in these cancer cells.

### TRIP-Br2 is aberrantly expressed in multiple human solid tumors and its overexpression is associated with poor prognosis in HCC

**Figure 5:**
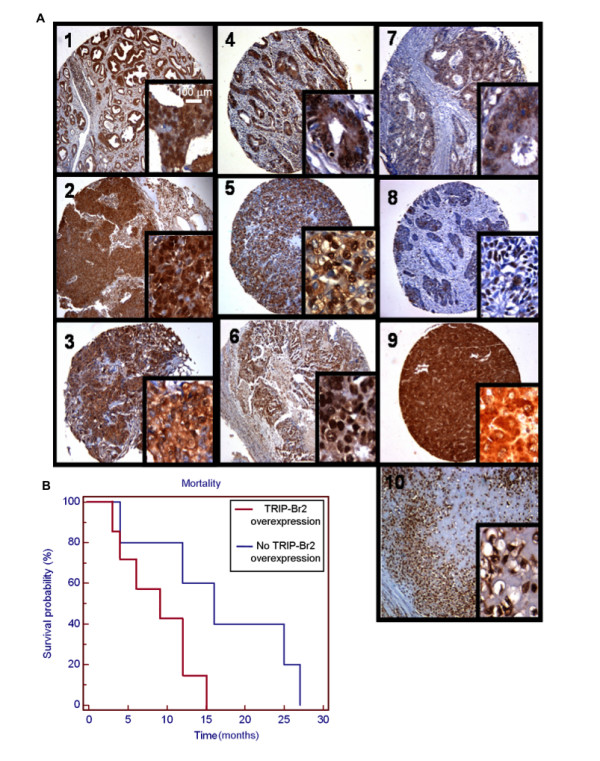
**TRIP-Br2 is overexpressed in multiple human solid tumors and associated with poor prognosis in hepatocellular carcinoma (HCC)**. (A) Multiple human tumor tissue arrays were immunostained with rabbit anti-TRIP-Br2 polyclonal antibodies. 1: Prostate carcinoma; 2: Squamous cell lung carcinoma; 3: Breast carcinoma; 4: Gastrointestinal stromal tumors, GIST; 5: Renal cell carcinoma; 6: Ovarian carcinoma; 7: Colon carcinoma; 8: Basal cell carcinoma; 9: Hepatocellular carcinoma; 10: Osteosarcoma. The small insert represents 400× magnification of the tissue in each window (shown at 100× magnification). A scale is included in the small insert of window #1 (for all 400× magnified tissue specimens). Immunopositive staining for hTRIP-Br2 is represented by the brown color against the hematoxylin (blue) counterstain. Data was obtained from three independent experiments that were performed in triplicates. (B) TRIP-Br2 overexpression is associated with poor survival of HCC patients (n = 12). The mean survival of patients with TRIP-Br2 overexpression (9 months) was significantly lower than that of HCC patients without TRIP-Br2 overexpression (16 months). The p-value of this survival analysis was determined to be 0.0452 using the Kaplan Meier log rank test.

In order to address an oncogenic role for TRIP-Br2 in human cancers, we assessed the immunohistochemical expression of TRIP-Br2 by comparing normal and cancer tissue sections on microarrays that were constructed from patient specimens of 10 different human tumor types. Tissue microarray (TMA) is a high-throughput method for the analysis of large numbers of formalin-fixed, paraffin-embedded (FFPE) materials with minimum cost and effort [31]. We found that TRIP-Br2 was overexpressed in prostate carcinoma (50.8%), squamous cell lung carcinoma (100%), lung adenocarcinoma (48.7%), ovarian cystadenocarcinoma (73.1%), colorectal carcinoma (64.9%), renal cell carcinoma (50%), osteosarcoma (100%) and hepatocellular carcinoma (72.4%). Notably, the frequency of TRIP-Br2 overexpression was lower in breast carcinoma (25%), basal cell carcinoma (16.7%) and gastrointestinal stromal tumor (15.6%). We also observed minor variations of TRIP-Br2 overexpression between different subtypes of ovarian carcinoma such as serous, mucinous and endometroid ovarian cystadenocarcinoma (data not shown). A representative TRIP-Br2-immunostained tumor specimen from each of the 10 tumor tissues and corresponding normal tissues examined by TMA are shown in Figure 5A and Additional File 2, respectively. The frequency of TRIP-Br2 upregulation in these human cancers is summarized in Additional Table S1 (see Additional File 1).

Next, we investigated the effect of TRIP-Br2 overexpression on the survival of hepatocellular carcinoma (HCC) patients to determine whether TRIP-Br2 overexpression is associated with poor prognosis. A patient cohort (n = 12) with full survival data was divided into two groups, survival ≤ 1 year (n = 8) and survival > 1 year (n = 4). These two groups were subsequently scored as "TRIP-Br2 overexpressors" versus "TRIP-Br2 non-overexpressors" in the corresponding tumor tissue biopsies represented on TMAs (Figure 5A). A patient was scored as a "TRIP-Br2 overexpressor" if the intensity of TRIP-Br2 immunostaining in tumor tissue was observed to be more intense than adjacent normal tissue. Six of eight HCC patients were TRIP-Br2 overexpressors and were found to have survived for ≤ 1 year, while three of four HCC patients were TRIP-Br2 non-overexpressors and were found to have survived for > 1 year. A survival analysis using the Kaplan Meier log rank test was performed, which showed that the mean survival of patients exhibiting tumor tissue TRIP-Br2 overexpression (9 months) was found to be significantly lower than the survival of HCC patients without evidence of TRIP-Br2 overexpression (16 months) (p = 0.0452) (Figure 5B). This observation is not only significant from a statistical viewpoint, but also clinically in the context of a cancer type with a particularly poor prognosis.

### RNA interference of TRIP-Br2 expression inhibits cell-autonomous growth of HCT-116 human colorectal cancer cells

**Figure 6:**
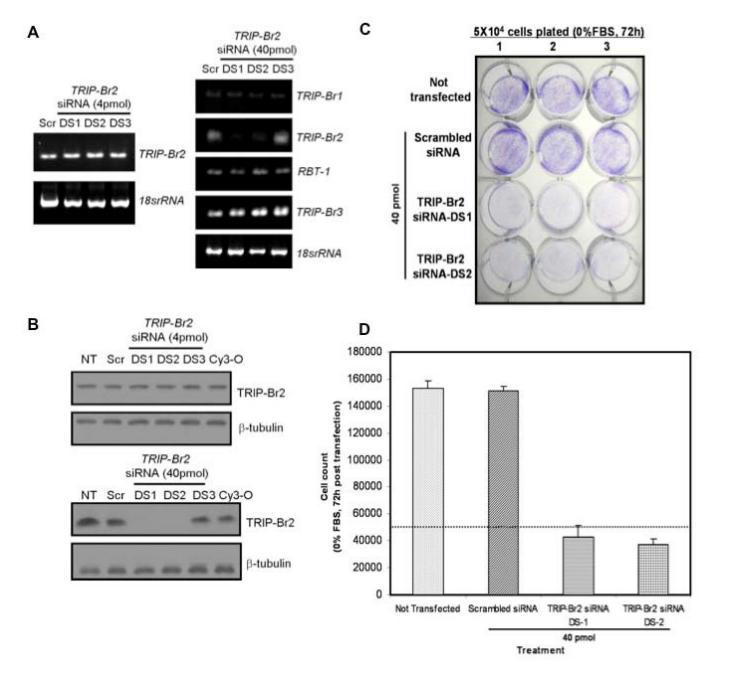
**siRNA knockdown of *TRIP-Br2 *expression inhibits cell-autonomous growth of HCT-116 cells**. 4 pmol or 40 pmol of Cy3-labeled oligomer control (Cy3-O), scrambled siRNA (Scr) and *TRIP-Br2-*specific siRNAs (DS1, DS2 or DS3) were transiently transfected into HCT-116 cells respectively. "Not transfected" (NT) samples were used as negative controls. The specificity of *TRIP-Br2*-specific siRNAs was assessed by semi-quantitative RT-PCR and Western blot analyses. Three independent experiments were performed in triplicates. (A) Knockdown of *TRIP-Br2 *expression in HCT-116 cells (12-well plate) was achieved by *TRIP-Br2-*specific siRNAs, DS1 and DS2, at the dose of 40 pmol. *18srRNA *was used as a loading control. (B) TRIP-Br2 protein expression was significantly knocked down by *TRIP-Br2*-specific siRNAs, DS1 and DS2, at the dose of 40 pmol. β-tubulin was used as a loading control. (C) Colony forming assays and (D) cell count analyses showed that siRNA knockdown of *TRIP-Br2 *expression (DS-1 or DS-2 at the dose of 40 pmol) inhibited cell-autonomous growth of serum-deprived HCT-116 cells. The dashed line indicates the initial cell density plated. Data was obtained from three independent experiments that were performed in triplicates.

To validate the potential of *TRIP-Br2 *as a novel transcription-based chemotherapeutic target for human cancers, we performed siRNA knockdown of *TRIP-Br2 *expression in HCT-116 cells. Cy3-labeled oligomer transfection control (Cy3-O), scrambled siRNA non-specific control (Scr) or *TRIP-Br2-*specific siRNAs (DS1, DS2 or DS3) were transiently transfected into HCT-116 cells, respectively, at a low dose of 4 pmol or a high dose of 40 pmol (in one ml of DMEM supplemented with 10% FBS). Twenty-four hours post-transfection, these cells were serum-deprived for 72 h to investigate the role of TRIP-Br2 in cell-autonomous growth of HCT-116 cells. As shown in Figure 6A (*Left panel*), specific knockdown of *TRIP-Br2 *expression in HCT-116 cells (12-well plate) was only achieved by *TRIP-Br2-*specific siRNAs, DS1 and DS2, at the higher dose of 40 pmol. There were no changes in the transcript levels of other *TRIP-Br *gene family members upon treatment with *TRIP-Br2-*specific siRNAs, DS1 and DS2, as assessed by semi-quantitative RT-PCR (Figure 6A, *right panel*).Western blot analyses further revealed that TRIP-Br2 protein expression was significantly knocked down by *TRIP-Br2*-specific siRNAs, DS1 and DS2 (Figure 6B). In addition, colony forming assays (Figure 6C) and cell count analyses (Figure 6D) showed that siRNA knockdown of *TRIP-Br2 *expression inhibited cell-autonomous growth of serum-deprived HCT-116 cells.

## Discussion

The TRIP-Br proteins represent a novel family of mammalian transcriptional coregulators that recruit PHD zinc finger- and/or bromodomain-containing transcription factors such as p300/CBP to the E2F/DP transcriptional complexes in order to regulate E2F-mediated gene transcription and cell cycle progression [7]. We recently reported that ablation of *TRIP-Br1 *or *TRIP-Br2 *expression suppresses serum-inducible *CYCLIN E *expression. The deficiency of either TRIP-Br1 or TRIP-Br2 resulted in proliferative block, indicating that these proteins may have interdependent but not superimposable roles in the regulation of serum-inducible cell cycle progression [17]. Although amplification of *TRIP-Br1 *is commonly detected in ovarian cancers [16] and overexpression of TRIP-Br1 has been shown to induce tumors in nude mice [18], the role of its closely related family member, TRIP-Br2, in cell cycle regulation and tumor progression has not been elucidated.

With an increasing number of mRNA expression profiling studies employing microarrays showing a positive correlation between *TRIP-Br2 *overexpression and cellular proliferation [32-37], we postulated that TRIP-Br2 plays an important protooncogenic role in cell cycle regulation and tumor progression. To validate its function(s) in growth and proliferation, we stably overexpressed TRIP-Br2 in NIH3T3 fibroblasts and demonstrated that TRIP-Br2 overexpression transformed these murine fibroblasts, rendering them capable of proliferation under low serum concentrations and of anchorage-independent growth in soft agar. We also demonstrated that overexpression of TRIP-Br2 induced tumors in athymic nude mice (nu/nu). Transformed cellular phenotypes were associated with dysregulation of the E2F/DP-transcriptional pathway through upregulation of a subset of key E2F-responsive genes, such as *CYCLIN E*, *CYCLIN A2, CDC6 *and *DHFR*. Furthermore, we have shown in our knockdown/knockout and overexpression studies that *CYCLIN E *is indeed a TRIP-Br-coregulated gene. Ongoing microarray studies will help us to identify other candidate TRIP-Br-coregulated genes and to establish the mechanism by which TRIP-Br proteins promote growth and tumor progression.

As overexpression of TRIP-Br2 resulted in the transformation of NIH3T3 fibroblasts, we hypothesized that TRIP-Br2 expression is dysregulated in human cancer. We found TRIP-Br2 to be overexpressed in many cancer cell lines and observed its localization to the nucleus. We subsequently showed that TRIP-Br2 was also overexpressed in many human cancers, including prostate carcinoma, squamous cell lung carcinoma, lung adenocarcinoma, ovarian cystadenocarcinoma, colorectal carcinoma, renal cell carcinoma, osteosarcoma and hepatocellular carcinoma. Notably, we observed that the expression pattern of TRIP-Br2 in these multiple human tumors *in vivo *matched that observed in cultured cells originally derived from these tumors. For instance, in both osteosarcoma tissues and U2OS cells, TRIP-Br2 was overexpressed and localized to the nucleus. No nuclear presence and little or no cytoplasmic expression of TRIP-Br2 were observed in normal prostate, lung, breast, gastric, ovary, colon, skin or kidney sections (Additional File 2). These data demonstrate that TRIP-Br2 is frequently and highly expressed in tumors, but not in the corresponding normal tissues and suggests that TRIP-Br2 expression and localization may be dysregulated in tumors. We have also observed overexpression of TRIP-Br2 in the cytoplasm of a small subset of these tumor specimens (data not shown), suggesting that TRIP-Br2 may perform novel functions in the cytoplasm and/or intracellular organelles to support oncogenesis in these tumor subsets. Collectively, our data suggest that *TRIP-Br2 *is a *bona-fide *protooncogene and that its overexpression may be associated with poor prognosis in human cancers, as demonstrated in the case of hepatocellular carcinoma.

We envisage that the mechanism of overexpression of TRIP-Br proteins may exist at the post-translational level in human cancers and may involve dysregulation of protein turnover. Indeed, we have recently shown that mutation of leucine residue 238 of the highly conserved nuclear export signal (NES) motif of TRIP-Br2 led to the nuclear entrapment of TRIP-Br2 and abolished it protein turnover [38]. Ongoing high-throughput DNA sequencing of the corresponding human tumor samples identified in our TMA immunoscreen will help us to identify novel disease-inducing mutations in the coding sequence, and the 5' and 3' regulatory regions of TRIP-Br2. We further validated the potential of *TRIP-Br2 *as a novel transcription-based chemotherapeutic target for human cancers by demonstrating that siRNA knockdown of *TRIP-Br2 *inhibited cell-autonomous growth of serum-deprived HCT-116 cells. Notably, we have also shown that antagonism of the TRIP-Br integrator function by synthetic decoy peptides, which compete with TRIP-Br for binding to PHD zinc finger- and/or bromodomain-containing proteins, arrests proliferation and induces caspase-3-independent sub-diploidization in cancer cells *in vitro *[23].

In summary, we have identified TRIP-Br2 as a novel protooncogene that is aberrantly overexpressed in human cancers. By making use of a comprehensive and high-throughput tissue microarray technology, we were able to advance rapidly from experimental validation of the protooncogenic role of TRIP-Br2 to identifying its value in translational medicine for the potential treatment of a wide variety of human cancers.

## Competing interests

The authors declare that they have no competing interests.

## Authors' contributions

All authors have read and approval the final manuscript. JKC participated in study design, data acquisition, interpretation and manuscript writing. LG participated in study design and data interpretation. ZZ, CY, SLN, KGS, JVB participated in data interpretation. XMS participated in tissue culture-related work. SAR and BKP participated in tissue microarray-related work. MST and SIH designed the study and led the data interpretation and manuscript writing.

## Supplementary Material

Additional file 1 **Additional Materials. **This file contains additional materials entitled 1) "Construction of C-terminal HA-tagged *hTRIP-Br2 *expression plasmid" (Additional Methods); 2) "Frequency of TRIP-Br2 overexpression in 10 different human cancers" (Additional Table S1); 3) "TRIP-Br2 expression in multiple normal human tissues and benign tumors" (Figure Legend for Additional Figure S1). Click here for file

Additional file 2 **TRIP-Br2 expression in multiple normal human tissues and benign tumors**. The data presents the results of immunostaining of multiple normal or benign human tumor tissue arrays with rabbit anti-TRIP-Br2 polyclonal antibodies. Click here for file
