# Supplementary material for: TRIP-Br2 promotes oncogenesis in nude mice and is frequently overexpressed in multiple human tumors
Source: J Transl Med. 2009 Jan 20;7:8. doi: 10.1186/1479-5876-7-8 (PMC2671481; doi:10.1186/1479-5876-7-8)
Supplement: Additional file 1 — Additional Materials. This file contains additional materials entitled 1) "Construction of C-terminal HA-tagged hTRIP-Br2 expression plasmid" (Additional Methods); 2) "Frequency of TRIP-Br2 overexpression in 10 different human cancers" (Additional Table S1); 3) "TRIP-Br2 expression in multiple normal human tissues and benign tumors" (Figure Legend for Additional Figure S1). [file 1479-5876-7-8-S1.doc]

**Additional Materials**

**TRIP-Br2 promotes oncogenesis in nude mice and is frequently overexpressed in multiple human tumors**

Jit Kong Cheong,^1,2^ Lakshman Gunaratnam,^1^ Zhi Jiang Zang,^1,2^ Christopher M Yang,^2^ Xiaoming Sun,^1^ Susan L Nasr,^1^ Khe Guan Sim,^2^ Bee Keow Peh,^3^ Suhaimi Bin Abdul Rashid,^3^ Joseph V Bonventre,^1^ Manuel Salto-Tellez,^3^ Stephen I Hsu^1,2^

^1^Renal Division and Department of Medicine, Brigham and Women’s Hospital and Harvard Medical School, Boston, MA 02115, USA

^2^Department of Medicine, National University of Singapore and National University Hospital. 119074, Singapore

^3^Department of Pathology, National University of Singapore and National University Hospital, 119074, Singapore

**Methods**

***Construction of C-terminal HA-tagged hTRIP-Br2 expression plasmid***

The nucleotide sequence of human *TRIP-Br2* (*hTRIP-Br2*) was obtained from NCBI PubMed (GenBank^TM^ accession no. BC101639) and used as the template for design of *hTRIP-Br2*-specific primers using DNAstar^TM^ software. These primers were modified to include the following features: a kozak sequence that precedes the 5’ end of the forward primers and an HA-tag coding sequence that follows the 3’ end of *hTRIP-Br2* reverse primers. Polymerase Chain Reactions (PCR) were performed on 1 µl cDNA samples, from WI38 human primary lung fibroblasts, in the presence of 10 mM deoxyribonucleotide triphosphates (dNTPs) and 10 µM of the above-mentioned primer pair in a total reaction volume of 20 µl. PCR was performed as follows: 30 cycles of denaturation (94^o^C, 30 seconds), annealing (56^o^C, 30 seconds) and extension (72^o^C, 90 seconds) with a 2-minute initial denaturation step at 94^o^C and a 3-minute terminal polishing step at 72^o^C. The amplicon was purified by QIAquick Gel Extraction kit (Qiagen, Inc., Valencia, CA) and cloned into pCR2.1®-TOPO® using the TOPO TA cloning® kit (Invitrogen, Carlsbad, CA), in accordance with the manufacturers’ instructions. The resultant plasmid was digested with Eco *RI* prior to ligation of the released insert into the same site in the pcDNA3.1 expression vector (Invitrogen, Carlsbad, CA). The resultant plasmid, pcDNA3.1-*hTRIP-Br2-HA*, was then transformed into *Escherichia coli* DH5α® hosts [genotype: φ80d*lacZ*ΔM15, *recA*1, *endA*1, *gyrA*96, *thi*-1, *hsdR*17 (r_k_^-^, m_k_^+^), *supE*44, *relA*1, *deoR*, Δ(l*acZYA-argF*) U169] (Promega Biosciences, Inc., San Luis Obispo, CA) and positive clones were selected by ampicillin (50 μg/ml, Sigma). Positive plasmid clones were further verified by Bam *HI* digestion, T7/HA-specific PCR as well as direct sequencing analysis of PCR products. Amplified PCR products and restriction enzyme-digested DNA samples were separated on 0.7% and 1% agarose gels respectively, and visualized by staining with ethidium bromide. The primer sequences used for molecular cloning are available upon request.

**Tables**

**Additional Table 1. Frequency of TRIP-Br2 overexpression in 10 different human cancers.**

| **Tissues** | **Cancer Types** | **Frequency of**  **TRIP-Br2 Upregulation**  **(% of tissue sections)** |
| --- | --- | --- |
| **Prostate** | **Prostate carcinoma (n=59)** | **50.8%** |
| **Lung** | **Adenocarcinoma (n=39)**  **Squamous cell carcinoma (n=19)** | **48.7%**  **100%** |
| **Breast** | **Invasive ductal carcinoma (n=86)** | **25.7%** |
| **Gastric** | **Gastrointestinal stromal tumors, GIST (n=32)** | **15.6%** |
| **Ovary** | **Ovarian cystadenocarcinoma (n=67)** | **73.1%** |
| **Colon** | **Colon carcinoma (n=37)** | **64.9%** |
| **Skin** | **Basal cell carcinoma (n=24)** | **16.7%** |
| **Kidney** | **Renal cell carcinoma (n=64)** | **50%** |
| **Bone** | **Osteosarcoma (n=3), full sections** | **100%** |
| **Liver** | **Hepatocellular carcinoma (n=29)** | **72.4%** |

**Figure Legends**

**Additional Figure 1.** TRIP-Br2 expression in multiple normal human tissues and benign tumors. Multiple normal or benign human tumor tissue arrays were immunostained with rabbit anti-TRIP-Br2 polyclonal antibodies. 1: Prostate (Normal); 2: Lung (Normal); 3: Breast (Normal); 4: Gastric (Normal); 5: Renal (Normal); 6: Ovarian cystadenoma (benign neoplasm); 7: Colon tissue (Normal); 8: Skin (Normal); 9: Liver tissue (Normal); 10: Bone tissue (Normal). The small insert represents 400× magnification of the tissue in each window (shown at 100× magnification). A scale is included in the small insert of window #1 (for all 400× magnified tissue specimens). Immunopositive staining for hTRIP-Br2 is represented by the brown color against the hematoxylin (blue) counterstain.
