# Supplementary figures and images for: TRIP-Br2 promotes oncogenesis in nude mice and is frequently overexpressed in multiple human tumors
Source: J Transl Med. 2009 Jan 20;7:8. doi: 10.1186/1479-5876-7-8 (PMC2671481; doi:10.1186/1479-5876-7-8)

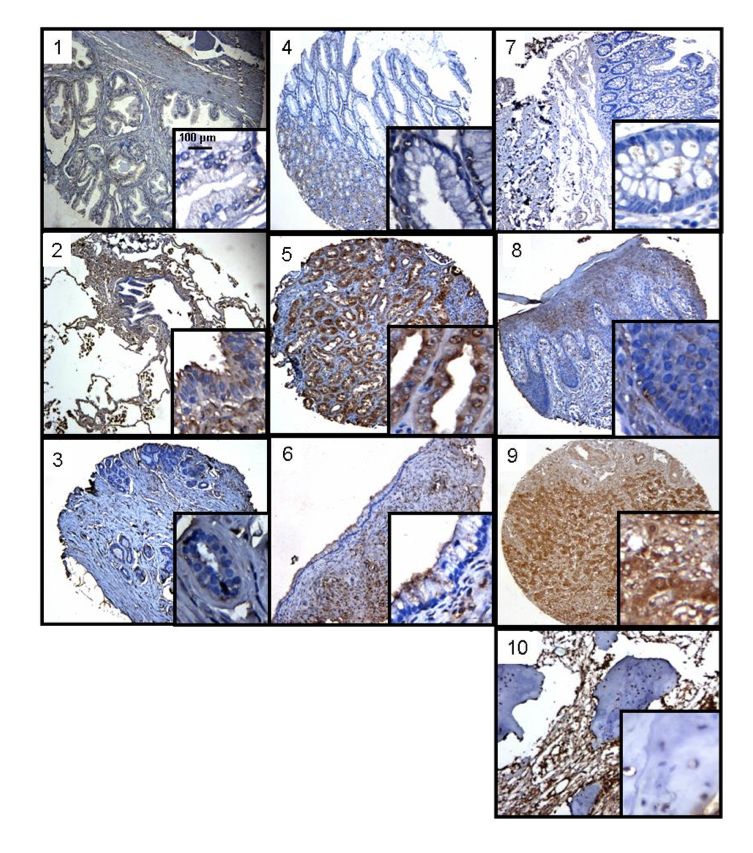

Supplement: Additional file 2 — TRIP-Br2 expression in multiple normal human tissues and benign tumors. The data presents the results of immunostaining of multiple normal or benign human tumor tissue arrays with rabbit anti-TRIP-Br2 polyclonal antibodies. [file 1479-5876-7-8-S2.tiff]
